# Individualized 3D printing navigation template for pedicle screw fixation in upper cervical spine

**DOI:** 10.1371/journal.pone.0171509

**Published:** 2017-02-02

**Authors:** Fei Guo, Jianhao Dai, Junxiang Zhang, Yichuan Ma, Guanghui Zhu, Junjie Shen, Guoqi Niu

**Affiliations:** 1 Department of Radiology, The First Affiliated Hospital of Bengbu Medical College, Bengbu, Anhui, China; 2 Department of Orthopedics, The First People's Hospital of Huainan, Huainan, Anhui, China; 3 Department of Orthopedics, The First Affiliated Hospital of Bengbu Medical College, Bengbu, Anhui, China; Shenzhen institutes of advanced technology, CHINA

## Abstract

**Purpose:**

Pedicle screw fixation in the upper cervical spine is a difficult and high-risk procedure. The screw is difficult to place rapidly and accurately, and can lead to serious injury of spinal cord or vertebral artery. The aim of this study was to design an individualized 3D printing navigation template for pedicle screw fixation in the upper cervical spine.

**Methods:**

Using CT thin slices data, we employed computer software to design the navigation template for pedicle screw fixation in the upper cervical spine (atlas and axis). The upper cervical spine models and navigation templates were produced by 3D printer with equal proportion, two sets for each case. In one set (Test group), pedicle screws fixation were guided by the navigation template; in the second set (Control group), the screws were fixed under fluoroscopy. According to the degree of pedicle cortex perforation and whether the screw needed to be refitted, the fixation effects were divided into 3 types: Type I, screw is fully located within the vertebral pedicle; Type II, degree of pedicle cortex perforation is <1 mm, but with good internal fixation stability and no need to renovate; Type III, degree of pedicle cortex perforation is >1 mm or with the poor internal fixation stability and in need of renovation. Type I and Type II were acceptable placements; Type III placements were unacceptable.

**Results:**

A total of 19 upper cervical spine and 19 navigation templates were printed, and 37 pedicle screws were fixed in each group. Type I screw-placements in the test group totaled 32; Type II totaled 3; and Type III totaled 2; with an acceptable rate of 94.60%. Type I screw placements in the control group totaled 23; Type II totaled 3; and Type III totaled 11, with an acceptable rate of 70.27%. The acceptability rate in test group was higher than the rate in control group. The operation time and fluoroscopic frequency for each screw were decreased, compared with control group.

**Conclusion:**

The individualized 3D printing navigation template for pedicle screw fixation is easy and safe, with a high success rate in the upper cervical spine surgery.

## Introduction

The difficult of posterior cervical spinal surgery is the focal point of this study. Nowadays, the primary system of internal screw placement in posterior cervical surgery is the screw-rod system[1], which includes pedicle screws system, lateral mass screws system, transarticular screws system, spinous screws system, and laminar screws system. The cervical pedicle screw internal fixation system is the product of spinal biomechanics, an area of study that is of milestone significance, laying the foundation for a greater understanding of the functional behavior and development of the spine. Clinically, the pedicle screw system has become the strongest and the most common internal fixation method for posterior cervical surgery because of its excellent biomechanical property [2].

The cervical pedicle screw internal fixation system is firmer and more stable than the lateral mass screws system. The study shows that the pull-out strength is twice that of the lateral mass screw [3]; thus, indications for its use are more extensive. Cervical pedicle screw internal fixation system can correct cervical deformity and instability, and can also restore and maintain normal intervertebral height and physiological curvature. As well, it provides obvious advantages for maintaining axial rotation of the cervical spine and flexion and extension stability [4, 5].

However, the traditional upper cervical pedicle screw fixation is difficult, inefficient, and carries a high risk of spinal cord and vertebral artery injury [6, 7]. Even experienced spine surgeons find quick, accurate screw placement difficult. The traditional surgical procedure uses repeated C-arm X-ray machine fluoroscopy during operation, which is time-consuming and increases the patient’s exposure to X-ray. Differences in individual upper cervical spine add the difficulty, making uniform screw placement problematic [8]. Deformity of the vertebral pedicle, variations in form, trend, symmetry, and width of vertebral pedicles, as well as a lack of local symmetry, can contribute to an unclear view of individual structural variations. Furthermore, large surgical navigation devices like CT 3D navigation system, G-arm, O-arm and other imaging equipments are expensive and not conducive to universal promotion.

Whether using the traditional free-hand technique or CT and digital computer navigation technique, the purpose is to reduce the risk, improve the success rate, and guarantee effective pedicle screw fixation [9]. In recent years, with the development of rapid prototyping technology, individualized 3D printing navigation template for pedicle screw fixation have become a hot spot in the spinal surgery [10–12]. In response, this study uses computer software and 3D printing technique to design and produce individualized 3D printing navigation templates for pedicle screw fixation in the upper cervical spine.

## Materials and methods

### Preparation of upper cervical spine models

The study was approved by the Ethics Committee of The First Affiliated Hospital of Bengbu Medical College. All the participants provided their written informed consent to participate in this study. At our imaging center, we collected continuous tomography data of cervical spine 64-slice spiral CT from 13 patients with upper cervical spine diseases. The scanning parameters were: voltage, 120 kV; electric current: 150 mA; layer thickness, 0.5 mm; screw pitch, 0.5 mm; matrix, 512×512. Original DICOM images were disposed with Mimics15.0. The cervical spine was segmented and extracted, and then models with STL format after 3D reconstruction were exported. An equal proportion of cervical spine was printed with the 3D printer, idea-Print F100, and PLA printing material. Print parameters were set to a single-story height of 0.18 mm, model wall thickness of 1.00 mm, filling rate of 15.0%, and filling speed of 100 mm/s.

### Preparation of individualized screw fixation navigation template

**Fig 1:**
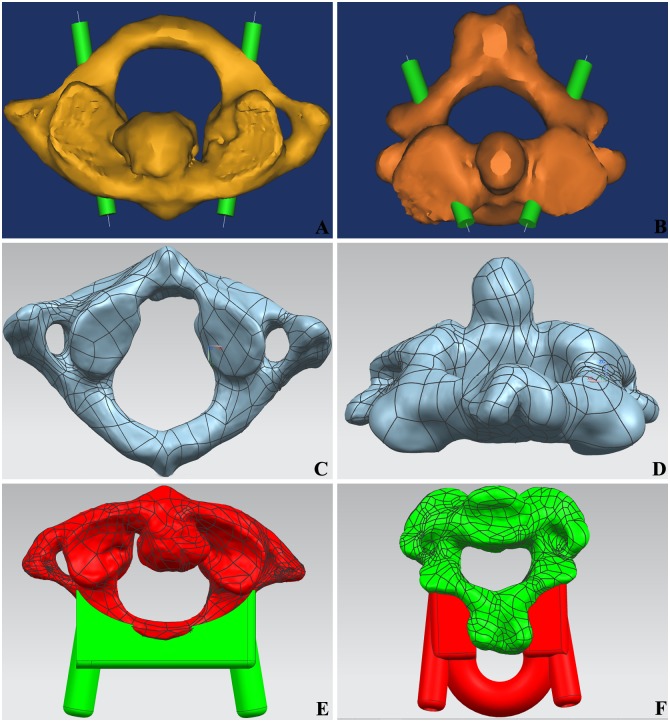
The design of individualized navigation templates for pedicle screw fixation. (A) Virtual axis through the pedicle of atlas. (B) Virtual axis through the pedicle of axis. (C) Grid mesh of atlas. (D) Grid mesh of axis; (E) The fitting condition of atlas navigation templates and vertebra; (F) The fitting condition of axis navigation templates and vertebra.

The virtual screws with a 4.0 mm diameter were designed with the MedCAD tool, *Mimics*. The optimal placement channel and entrance point were obtained through minor adjustments to ensure that the screws were located within long axis of the vertebral pedicle and to ensure that no walls pierced. A straight line was introduced in the virtual screw center, then saved and exported it in Initial Graphics Exchange Specification (IGS) format (Fig 1A and 1B). The 3D models from the single upper cervical spine (atlas and axis) were exported and saved in Standard Template Library (STL) format. The accurate surface processing of cervical vertebra was performed with triangular mesh in Geomagic Studio 2013 Software, and then saved the data in IGS format (Fig 1C and 1D).

The 3D reconstruction models of upper cervical vertebra were opened by NX 8.5 software. The spinous process, the transverse process and the vertebral arch lamina in upper cervical spine were extracted. The reverse templates consistent with the anatomical form of above osseous tissues were designed. The IGS format line was exported into NX8.5 as a reference and established 3D coordinate system. A concentric cylinder centered on the format line was designed in the 3D coordinate system. The internal diameter was 4 mm, and the external diameter was 8 mm. The virtual cervical screws in vertebral pedicle were adjusted to the best position. The length of screw was measured with a measuring tool in NX 8.5 software, and this provided reference for the depth of pedicle screw fixation. After subtracting from the vertebral body, the reverse template was summated with the cylinder, and finally formed the navigation templates STL files (Fig 1E and 1F).

**Fig 2:**
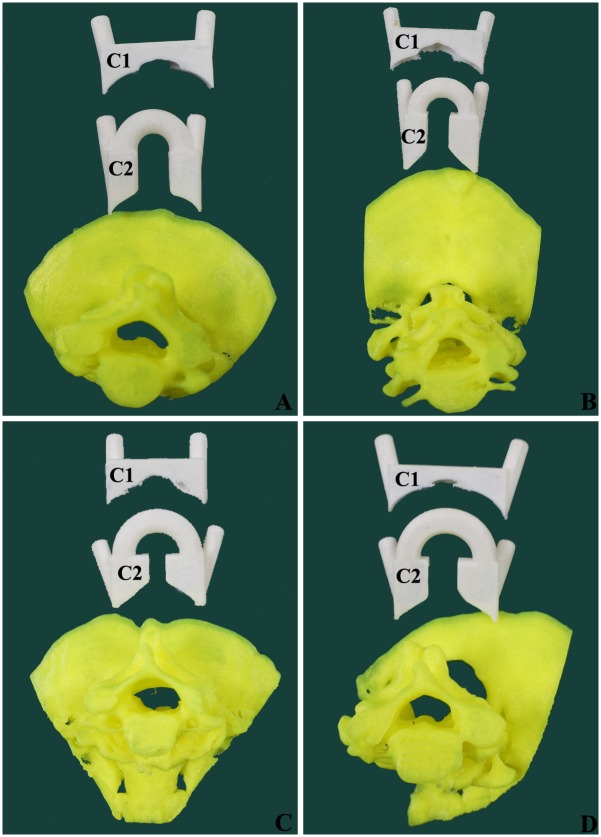
The cervical spine models and navigation templates printed by 3D printer. (A) Mode from a female patient aged 32 years with atlantoaxial dislocation. (B) Mode from a male patient aged 57 years with atlantoaxial dislocation. (C) Mode from a female patient aged 50 years with atlantoaxial dislocation. (D) Mode from a male patient aged 36 years with atlantoaxial dislocation.

The navigation template STL files were opened by idea-Print F100, which has its own processing software, ideaMaker. The navigation template of upper cervical spine was printed using the following print parameters: single slice height, 0.1 mm; model wall thickness, 2.0 mm; filling rate, 25%; filling speed, 100 mm/s. The base and external supports were also printed (Fig 2).

### Simulated operation of upper cervical pedicle screw fixation

A 3D printer then produces two sets per case of equal proportion. In the test group, pedicle screw fixation was guided by navigation template; in the control group, the screws were fixed under C-arm X-ray fluoroscopy. Both the operations were operated by the same chief physician of spinal surgery from our hospital.

**Fig 3:**
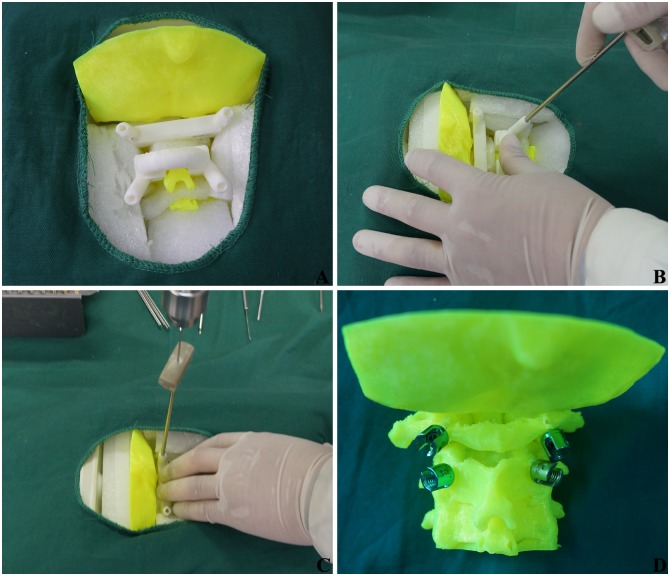
Navigation template-assisted pedicle screw fixation in upper cervical spine. (A) Navigation template was pressed to fit with vertebral. (B) The T-navigation protective sleeve was placed. (C) Kirschner wire perforated along protective sleeve. (D) Pedicle screws fixation were finished.

Fig 3 illustrates the navigation template-assisted pedicle screw fixation. The 3D cervical spine model was placed in a container with a large size and good X-ray penetration. The lacuna was filled with Polystyrene foam block. The dorsal bony structure was exposed in the prone position, simulating surgery. Navigation templates were pressed to fit with the related dorsal vertebral plate, facet joint, spinous process and posterior arch; the assistant maintained the pressure throughout the whole procedures. After the drill bit’s T-navigation protective sleeve was arranged on the navigation template cylinder, a 2.0 mm-diameter Kirschner wire was used to drill along the navigation protective sleeve. When the Kirschner wire got into bone cortex, the entrance point and direction were adjusted with lateral perspective C-arm X-ray fluoroscopy, and continued, ensuring the walls were unbroken. When the depth of Kirschner wire was closed to the length of screw per virtual measurement software, the navigation template was removed, the hole was expanded with open cone, tapped with a screw tap, and a 3.5 mm-diameter screw was inserted. All the screw positions were checked and treated with lateral perspective fluoroscopy. The operation time and fluoroscopic frequency for each screw were recorded.

**Fig 4:**
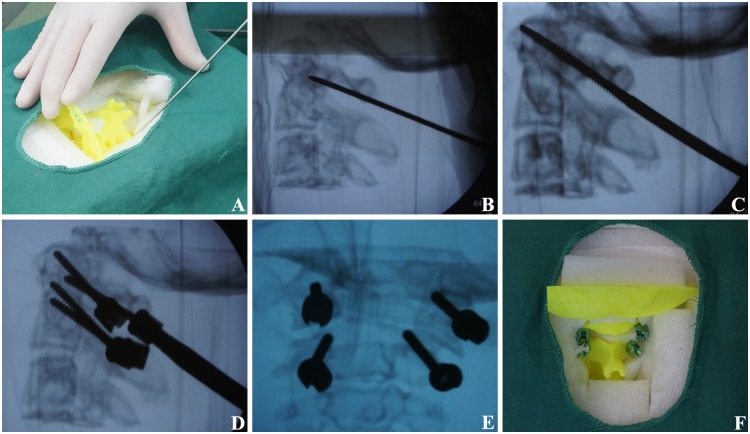
Pedicle screw fixation under C-arm X-ray machine fluoroscopy. (A) The entrance point was determined according to anatomic marker. (B) The direction of long axis of vertebral pedicle were explored under fluoroscopy. (C) The optimal placement channel was explored under fluoroscopy. (D) The entry angle was adjusted under fluoroscopy. (E) Frontal radiograph was adopted after screw fixation; (F) Pedicle screw fixation was finished.

Fig 4 illustrates the pedicle screw fixation under fluoroscopy. The 3D cervical spine model was fixed as above. The entrance point was determined according to anatomic marker. The bony cortex was penetrated by an open cone at the entrance point and repeated frontal and lateral fluoroscopy was performed by a C-arm X-ray machine to explore the long axis direction of the vertebral pedicle, until the walls around the hole were continuous. A 2.5 mm-diameter screw tap moved slowly under the fluoroscopy until the optimal placement channel was found. Then the upper cervical pedicle screw was slowly fixed into place, with repeated frontal and lateral fluoroscopy throughout the process to modify any possible deviation. The operation time and fluoroscopic frequency for each screw was also recorded.

### Evaluation of upper cervical pedicle screw fixation

**Fig 5:**
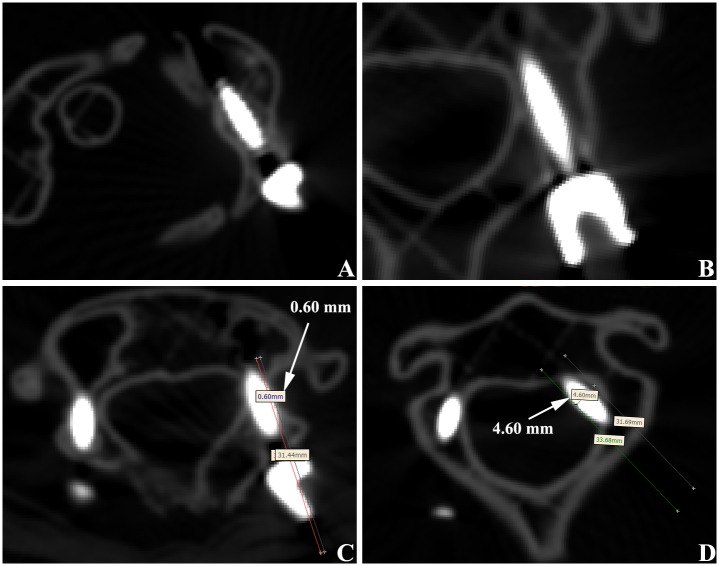
CT evaluation of pedicle screw fixation in upper cervical spine. (A) Atlas pedicle screw was totally in vertebral pedicle, which was considered as type I. (B) Axis vertebral pedicle screw was totally in vertebral pedicle, which was considered as type I. (C) Atlas pedicle screw broke lateral cortex of pedicle with a distance of 0.60 mm, which considered as type II. (D) Axis vertebral pedicle screw broke lateral cortex of pedicle with a distance of 4.60 mm, which was considered as type III.

The cervical spine models were performed in a 64-slice spiral CT scanning after pedicle screw fixation. Accuracy of the screw fixation was evaluated with Mimics15.0 software. The data was measured at all the levels of each screw. The final data was based on the lowest level of screw fixation, and the average results were calculated from measurements taken three times. The fixation effects were divided into 3 types [13, 14], according to the degree of pedicle cortex perforation and whether it needed to be renovated: Type I, screw is fully located within the vertebral pedicle; Type II: degree of pedicle cortex perforation is <1 mm, but with good internal fixation stability and no need to renovate; Type III, degree of pedicle cortex perforation is >1 mm or with poor the internal fixation stability and in need of renovation (Fig 5). Type I and Type II were acceptable placements; Type III placements were unacceptable.

### Statistical method

The data of the test group and control group were analyzed with SPSS 17.0 software. We tested statistical differences of screw placement accuracy with chi-square test. Statistical differences on quantitative data of the screw placement was compared by paired *t*-test (inspection level: α = 0.05)

## Results

Reconstructed data of this study were derived from 13 patients with upper cervical disease, and consisted of 6 males and 7 females, aged 25years to 57 years. Detailed information was shown in Table 1, which includes six cases of basilar invagination, four cases of atlantoaxial dislocation, two cases of atlas fracture and one case of tumor in vertebral canal.

**Table 1:** Basic data of 13 patients with upper cervical spine disease.

Number	Sex	Age (Y)	Diagnosis	Template	Screws
1	F	32	atlantoaxial dislocation	C1, C2	4
2	F	50	atlantoaxial dislocation	C1, C2	4
3	M	36	atlantoaxial dislocation	C1, C2	4
4	M	57	atlantoaxial dislocation	C1, C2	4
5	F	44	atlantoaxial dislocation, basilar invagination	C2	2
6	M	45	atlantoaxial dislocation, basilar invagination	C2	2
7	M	52	atlantoaxial dislocation, basilar invagination	C2	2
8	F	44	atlantoaxial dislocation, basilar invagination	C2	2
9	M	50	tumor in vertebral canal	Right C2	1
10	F	25	atlantoaxial dislocation, basilar invagination	C2	2
11	M	55	atlas fracture	C1, C2	4
12	F	44	atlantoaxial dislocation, basilar invagination	C2	2
13	F	52	atlas fracture	C1, C2	4

According to the reconstructed data, 13 pairs of upper cervical spine models, with two of each case printed; 19 navigation templates were printed, including 6 bilateral atlas navigation templates, 12 bilateral axis navigation templates and a right axis navigation template were printed. Atlas deformation in 6 cases with basilar invagination was serious and failed to produce a navigation template. Bilateral atlas and left axis in a case with in intraspinal tumor were severely damaged and failed to produce a navigation template.

A total of 74 cervical pedicle screws were placed in 2 groups. Of the 37 screws in test group, 32 were type I (86.49%), 3 were type II (8.11%), and 2 were type III (5.40%). Of the 37 screws in control group, 23 were type I (62.16%), 3 were type II (8.11%), and 2 were type III (29.73%). The acceptable rates of screw fixation in the test group and control group were 94.60% and 70.27%, respectively, while the unacceptable rates were 5.40% and 29.73%, respectively. The acceptable rates for the test group was significantly higher than that of the control group (χ^2^ = 7.703, *P* = 0.021).

There was no significant difference in the length of screw fixation between the test group and control group. The average operation time of screw fixation in the test group was 10.73 min, and the average fluoroscopic frequency was 10.95 times. The average operation time of screw fixation in control group was 27.70min, and the average fluoroscopic frequency was 40.35 times. Compared with control group, the operation time and fluoroscopic frequency for each screw decreased significantly (Table 2).

**Table 2:** Comparison of screw length, fixation time and perspective times between two test group and control group.

Group	screw length (mm)	operation time (min)	fluoroscopic frequency (number)
Test group	28.38±2.98	10.73±2.17	10.95±1.74
Control group	28.65±2.83	27.70±5.38	40.35±7.65
*t*	2.237	10.547	13.512
*p*	0.815	<0.001	<0.001

## Discussion

Due to the anatomical characteristics of upper cervical pedicle, the posterior pedicle screw fixation is difficult and risky, and the consequences are often extremely serious if the operation fails or screw fixation is poor [15]. Because of the special vertebral artery course in C2, the C2 cervical foramina was closer to the interior than other cervical vertebra and clings to C2 external wall of vertebral pedicle, forming a depression or notch. The pedicle section is "C" type, which lead to the weak area of C2 vertebral pedicle and is easily injured when placing pedicle screw.

When establishing the pedicle screw channel during the operation, if screw fixation direction cannot be grasped well, the spinal cord or vertebral artery can be hurt, and internal fixation strength may decline or lose efficacy [16]. Common methods of the upper cervical pedicle screw fixation are the free-hand technique, the imaging perspective fixation method, the 3D CT navigation and positioning method, and the 3D printing navigation template fixation method. The free-hand technique is the oldest and least commonly used. Its increased risk of screw fixation complication is lactated to the experience of the surgeons, with a failure rate ranging from 3% to 55% [17–19].

The imaging perspective fixation method uses the developing bony anatomy as a reference point to determine the entrance location and adjust the needle-puncturing angles using intraoperative X-ray [20]. The method requires a surgeon with a wealth of experience, accurately combining radiographic images. Patients with upper cervical deformity, the organizational structure is chaotic, the development is not clear, and screw fixation is difficult. To ensure the safe screw placement, this method requires repeated frontal and lateral perspective, which increases operation time, more bleeding in surgery, more patients and surgical staff exposure to X-ray. Frequently moving the X-ray machine and repeated entering and leaving the operation room may also increase the chance of surgical infection. The 3D CT navigation and positioning method cannot gain 3D image data and real-time computer reconstruction data [21–23]. The entrance point and direction are guided in real time. Accurate intraoperative navigation and positioning are provided. The system is expensive and requires more personnel to participate, which general hospital usually cannot provide.

In this study, individualized 3D printing navigation templates which assist pedicle screw fixation in the upper cervical spine are developed by medical imaging, digital 3D reconstruction technology, reverse engineering, and rapid prototyping technology The use of navigation templates is simple and convenient. There are bilateral pedicle screw fixation holes in the navigation templates, so location failure will not occur due to position change. The operation is simple, and requirement of the operation are not high. The essentials of screw fixation can be mastered in a short time to complete the fixation.

In our study, 37 upper cervical screws were placed with the imaging perspective fixation method and the navigation template method. The acceptable placement rate with the navigation template method was 94.60%, while the acceptable rate with imaging perspective fixation method was 70.27%. The difference was statistically significant. Screw fixation using navigation templates only needs X-rays at the beginning and the end of the screw placement. This greatly reduces the X-ray exposure rates of the surgeon and the patients. This study shows that the operation time of navigation template method is cut by more than half that of the imaging perspective method, and the X-ray exposure time is cut to about quarter of that of the imaging method, which is safer for both the doctors and patients.

Individualized 3D printing navigation template-assisted pedicle screw fixation in upper cervical spine is low cost and does not need special equipment. There are no special requirements of the hospital and no need for special operating room instruments, so non-specialized hospital can provide this service. However, this method does require that the surgeon be proficient in the operation of Mimics, Geomagic, NX and other computer software, and the learning curve is long. The process of image data reconstruction, navigation template design and printing, a small number of errors may occur, which may be the reason why 5% of patients in this study resulted in a Type III screw placement. Certain disease of some patients in this study destroyed the anatomical structure of the posterior vertebral arch. Therefore, the navigation templates for some patients were not made successfully, and pedicle screw fixation could not be carried out, which is an influence of the disease rather than the method. During the operation, the assistant is required to buckle the templates and the vertebral body closely. Kirschner wire penetrates quickly and reduces the possibility of slippage between the template and the vertebral body.

## Conclusion

In this study, individualized 3D printing navigation templates for pedicle screw fixation in the upper cervical spine are designed through the union of medical imaging, digital 3D reconstruction technology, reverse engineering and rapid prototyping technology. Our results concluded that the individualized 3D printing navigation template for pedicle screw fixation is easy and safe, and has a high success rate for surgery in the upper cervical spine.

## Supporting information

S1 File The relevant data about this manuscript. (XLS)
