# Correction: Individualized 3D printing navigation template for pedicle screw fixation in upper cervical spine

**DOI:** 10.1371/journal.pone.0212213

**Published:** 2019-02-07

**Authors:** The PLOS ONE Editors

The authors advised that there is an error in the Abstract of this article [1]. The sentence beginning, “A total of 19 upper cervical spine and 19 navigation templates,” should instead read, “A total of 13 upper cervical spine and 19 navigation templates.”

This updated information aligns with the information provided in the Results section:

“According to the reconstructed data, 13 pairs of upper cervical spine models, with two of each case printed; 19 navigation templates were printed, including 6 bilateral atlas navigation templates, 12 bilateral axis navigation templates and a right axis navigation template were printed.”

## References

[1] Guo F, Dai J, Zhang J, Ma Y, Zhu G, Shen J, et al (2017) Individualized 3D printing navigation template for pedicle screw fixation in upper cervical spine. PLoS ONE 12(2): e0171509 10.1371/journal.pone.0171509 28152039 PMC5289602

